# A Centenary Collapse

**Published:** 1892-06-25

**Authors:** 


### Passing Topics.

### A CENTENARY COLLAPSE.

The condition of the Royal Sea Bathing Infirmary for Scrofulous Patients at the present moment is nothing less than a scandal to a community which takes credit for its philanthropy. If the Infirmary were an institution purely local in work and character, it is probable that its roots would have entered deeply enough into the soil on which it stands to ensure its proper nutrition. As it is, the institution, although at Margate, is not of Margate, and the well-to-do population of the metropolis and the other large towns it chiefly benefits appears quite content that being out of sight it shall prove to be effectually out of mind.

The Infirmary contains 220 beds, and has recently completed its centenary, when, we believe, a special effort was made to retrieve its financial position, which for some years past has been going steadily from bad to worse, until every penny of capital has been expended, and the Committee must now perforce sue *in formâ pauperis*.

But the effort, such as it was—whether skilfully and industriously made we have no means of judging—has lamentably failed, and the climax has been reached. A fiat has gone forth that the none too great accommodation of the Infirmary is to be diminished by no less than 140 beds, the Committee hoping by this drastic means to stay the downward course.

Now, here we have a history where, incidentally, a very serious reflection is cast upon the merits of that unaided method of voluntary support for our medical charities, which we all nevertheless desire to maintain if only it can be purged of its eccentricities. An institution patently useful to the whole kingdom, if not actually indispensable, and against whose management, so far as we know, nothing is alleged, is virtually to fade out of existence for want of the help it is nobody's, or rather it is everybody's, business to render. We can conceive of few stronger cases than that the Royal Sea Bathing Infirmary is enabled to put forward. The institution collects within its walls, from all parts of the country, a class of patients suffering from a terrible form of malady, often hereditary and not seldom incapacitating, and we are told upon undoubted authority that the results of treatment in the peculiarly pure and bracing atmosphere the Kentish Foreland boasts, aided by the beneficent action of the ocean, are such as "cannot be achieved elsewhere."

Where, we may ask, sleeps the philanthropy which is not awakened by so forcible an appeal? Alas! when many applicants cry for help, some must be overlooked, and as with individuals so with institutions; the most deserving may fail, where those who are merely bold succeed. This is a result for which a remedy ought to be found. Beneficence itself wearies of over-solicitation, and the mingling of cries that never cease, produces a distraction which in time settles into indifference. Possibly the collapse of the Margate Infirmary would not be an unmixed evil if it helped to draw attention to the great and growing difficulties under which much useful work is carried on. But the experience is too costly to be bought by means of an experiment, and we must hope that not only the danger to one meritorious institution may be averted by timely pleading on its behalf, but that certain general deductions will be drawn to the advantage of other and almost equally needy charities.

Agricola.

